# Comprehensive Molecular Profiling of Archival Bone Marrow Trephines Using a Commercially Available Leukemia Panel and Semiconductor-Based Targeted Resequencing

**DOI:** 10.1371/journal.pone.0133930

**Published:** 2015-07-29

**Authors:** Stephan Bartels, Elisa Schipper, Hans Heinrich Kreipe, Ulrich Lehmann

**Affiliations:** Institute of Pathology, Hannover Medical School, Hannover, Germany; University of Texas MD Anderson Cancer Center, UNITED STATES

## Abstract

Comprehensive mutation profiling becomes more and more important in hematopathology complementing morphological and immunohistochemical evaluation of fixed, decalcified and embedded bone marrow biopsies for diagnostic, prognostic and also predictive purposes. However, the number and the size of relevant genes leave conventional Sanger sequencing impracticable in terms of costs, required input DNA, and turnaround time. Since most published protocols and commercially available reagents for targeted resequencing of gene panels are established and validated for the analysis of fresh bone marrow aspirate or peripheral blood it remains to be proven whether the available technology can be transferred to the analysis of archival trephines. Therefore, the performance of the recently available Ion AmpliSeq AML Research panel (LifeTechnologies) was evaluated for the analysis of fragmented DNA extracted from archival bone marrow trephines. Taking fresh aspirate as gold standard all clinically relevant mutations (n = 17) as well as 25 well-annotated SNPs could be identified reliably with high quality in the corresponding archival trephines of the training set (n = 10). Pre-treatment of the extracted DNA with Uracil-DNA-Glycosylase reduced the number of low level artificial sequence variants by more than 60%, vastly reducing time required for proper evaluation of the sequencing results. Subsequently, randomly picked FFPE samples (n = 41) were analyzed to evaluate sequencing performance under routine conditions. Thereby all known mutations (n = 43) could be verified and 36 additional mutations in genes not yet covered by the routine work-up (e.g., *TET2*, *ASXL1*, *DNMT3A*), demonstrating the feasibility of this approach and the gain of diagnostically relevant information. The dramatically reduced amount of input DNA, the increase in sensitivity as well as calculated cost-effectiveness, low hands on , and turn-around-time, necessary for the analysis of 237 amplicons strongly argue for replacing Sanger sequencing by this semiconductor-based targeted resequencing approach.

## Introduction

Comprehensive mutation profiling in the routine work-up of samples from patients with a myeloid malignancy becomes more and more important. It increases the diagnostic accuracy, contributes to risk stratification and proposes also new therapeutic options [1]. Since sequencing costs dropped dramatically due to the introduction of next generation sequencing technologies [2] and bench-top platforms affordable for many laboratories entered the market it is now feasible to analyze a panel of around 30 genes in 10 samples within a few working days with reagent costs of much less than 1000 Euro per sample. Therefore, research laboratories as well as companies have started to develop and validate gene panels for routine diagnostics of myeloid malignancies [3,4]. However, nearly all of the published studies are based on the analysis of freshly collected peripheral blood samples or bone marrow aspirates and commercially available systems are optimized for these sample types which provide abundant amounts of high-quality high molecular weight genomic DNA [5,6].

For the diagnosis of many bone-marrow derived hematological diseases the morphological evaluation of formalin-fixed decalcified bone-marrow trephines represent the gold-standard because they provide superior morphological details and enable extensive immunohistochemical characterization of all cell types within the topological context of the bone marrow. This is especially important in case of developing bone marrow fibrosis, a finally lethal complication of many hematological malignancies, because under these circumstances bone marrow aspirates are often hypocellular or even acellular and not representative (i.e., *punctio sicca*). Therefore, the applicability of existing hematological gene panels and protocols for the evaluation of bone-marrow trephines has to be evaluated.

In the present study we tested the recently available AML Research panel from Life Technologies/ThermoFisher Scientific (Carlsbad, CA, USA) in a series of paired fresh frozen aspirates and fixed decalcified trephines taken the very same day. Despite its long development time involving several research groups and its release in July 2014 only a single poster abstract authored by employees from LifeTechnologies/ThermoFisherScientific reporting on the analysis of 4 peripheral blood samples could be identified as of March 23^th^ 2015 (AACR proceedings 2014). Therefore, we initiated in a first step an evaluation of the performance and quality parameters of the AmpliSeq AML Research panel in a series of 10 freshly collected bone marrow aspirates. The amplicon-based AML panel includes hot spots and complete coding regions of 19 genes relevant in hematopathology. The panel allows among other markers the analysis of the complete coding regions of the important hematologic marker genes *DNMT3A* and *TET2*, as well as the large exon 12 of *ASXL1*, which is an independent marker for adverse outcome in different myeloid malignancies [7,8]. In a second step, in order to cope with the well-described background of formalin-induced sequence changes which may lead to false-positive results [9] in the diagnostic setting two different DNA isolation protocols employing UNG glycosylase were evaluated and compared with a standard procedure.

Finally, the usefulness and robustness of the NGS protocol established within the training series of paired samples were subsequently evaluated in a series of 41 cases under routine conditions (including estimation of reagent costs per sample and turn-around-time).

## Materials and Methods

### Patient samples

From the archive of the Institute of Pathology DNA samples isolated from unfixed bone marrow aspirates were identified for which also a fixed, decalcified and embedded trephine taken the very same day exists. Patient samples were retrieved retrospectively in a completely anonymized fashion following the guidelines of the local ethics committee (“Ethics committee of the Medical School Hannover/Ethik-Kommission der Medizinischen Hochschule Hannover", head: Prof. Dr. Tröger). Due to the completely anonymized retrieval of the samples the ethics committee waived for the project described in this manuscript the need for individual informed consent for every single sample included and approved the study in its present form.

Selection criteria were availability and amount of DNA left over from the routine diagnostic procedures. The 10 samples meeting these criteria represent a spectrum of myeloid malignancies: 1x RARS, 4x RAEB-1, 1x RAEB-2, 2x CMML-1, and 1x AML M4 Eo.

For validation of the NGS approach under routine conditions with fixed, decalcified and embedded trephines, 41 archival bone marrow biopsies from 2000 till 2015 with known pathogenic mutations were selected following the ethics guidelines described in the preceding paragraph. The samples represent a spectrum of myeloid malignancies: AML, PV, PMF, RCMD, and RT.

### DNA isolation

**Fig 1:**
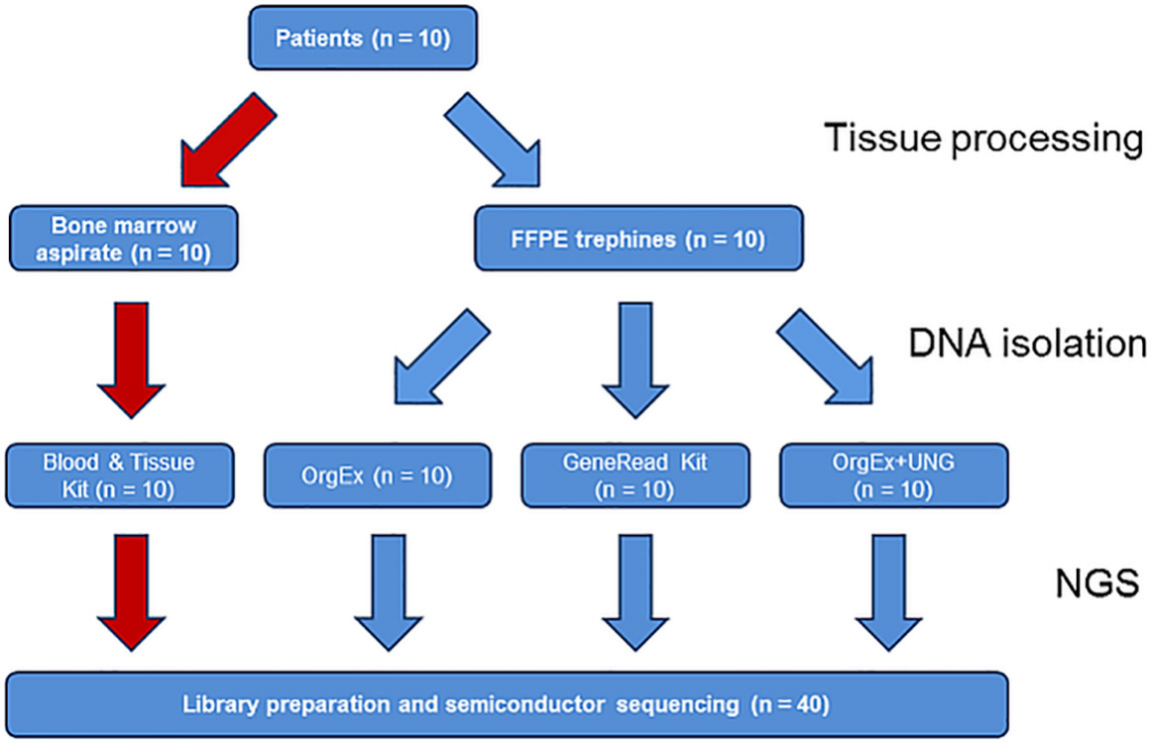
Flow diagram of sample processing from the training cohort.

DNA from fresh bone marrow aspirates was isolated with DNeasy Blood and Tissue Kit (Qiagen, Hilden, Germany). From the FFPE blocks 15 μm thick sections were collected, 5 sections each for the three extraction protocols: a) a standard proteinase K digestion followed by exhaustive organic extraction [10], b) a standard proteinase K digestion followed by exhaustive organic extraction with subsequent Uracil-DNA-Glycosylase treatment, and c) GeneRead DNA FFPE Kit (Qiagen, Hilden, Germany) according manufacturer´s instructions. Fig 1 shows a flow diagram of the sample processing. DNA quantification was performed using the Qubit 2.0 Fluorometer with Qubit *dsDNA HS* (*High Sensitivity*) *Assay Kit* (Life Technologies, Carlsbad, CA, USA)

### Uracil-DNA-Glycosylase (UNG) treatment

150 ng of DNA isolated by the standard protocol were incubated with 5 units UNG from Fermentas (Waltham, MA, USA) in a final volume of 20 μl. The incubation was performed in low binding reaction (1.5 ml DNA LoBind Tubes from Eppendorf, Hamburg, Germany) in a thermoblock for 2 h at 37°C and 10 min at 95°C. Following UNG treatment fluorimetric DNA quantification was repeated as described above.

### Ion AmpliSeq AML Panel Research panel

The Ion AmpliSeq AML Research Panel (Life Technologies, Carlsbad, CA, USA) comprises 237 amplicons from 19 genes which are well-described to be of relevance in myeloid malignancies, especially acute myeloid leukemia (see S1 Table). The panel consists of 4 primer pools; each requires 10 ng of DNA input material. Amplicon lengths were between 67 and 215 bp.

Due to patent protection of the diagnostically relevant *FLT3-ITD* this region is not covered by the amplicon design.

### Semiconductor-based targeted resequencing

Library preparation was performed with Ion AmpliSeq Library Kit 2.0. Quantification of prepared libraries was conducted by qPCR using the Ion Library Quantification Kit. For template preparation using the Ion OneTouch 2 instrument 6 patient samples were pooled (100 pM each). Sequencing was performed with Ion PGM Sequencing 200 Kit v2 on 318 v2 Chips.

### Bioinformatics

Analyses of sequencing raw data were performed with Torrent server software (Version 4.2.1), IGV-Browser (Version 2.3.34) and Cartagenia Bench Lab NGS software (Version 4.0). Parameters for analysis exclude single nucleotide variants with an allele frequency <2% and complex mutations with an allele frequency <5%, and a quality score (PHRED-scaled probability of incorrect calls) below 100.

### Sanger sequencing

For Sanger sequencing PCR amplification of DNA was done in a total volume of 25 μl PCR mix containing 10–50 ng template DNA, Taq buffer, 2.5 mM MgCl_2_, 200 μmol of each deoxynucleotide triphosphate, 10 pmol of each primer, 0.5 U of Platinum Taq (Invitrogen). PCR amplification conditions: 95°C 10 min; 95°C 30 sec, 60°C 45 sec, 72°C 30 sec for 40 cycles; 72°C 10 min. Subsequently the PCR products where purified with GenUP PCR Cleanup Kit (Biotechrabbit, Henningsdorf, Germany) and sequencing reaction where performed with 4 μl GenomeLab DCTS Quick Start Mix (Beckman Coulter, Brea, CA, USA), 1 μl Primer (5 pmol/μl) and 10–50 ng purified PCR product. Fluorescence labelled reaction products were analyzed utilizing a GenomeLab GeXP capillary sequencer (Beckman Coulter, Brea, CA, USA). The sequence data files were analyzed with SeqMan Pro software version 8.1.4 (DNASTAR).

#### Pyrosequencing

Pyrosequencing analysis was performed as described [11]. For each sample, 10 ng of genomic DNA were amplified with primer pairs for *JAK2* Codon 617, *IDH1* Codon 132, *KRAS* Codon 12/13, and *NRAS* Codon 12/13. For each region forward and reverse strand were analyzed independently. Each PCR product was analyzed by Pyrosequencing using PyroMark Gold Q96 reagents (Qiagen, Hilden, Germany), and Streptavidin Sepharose High Performance (GE Healthcare Bio-Science AB, Uppsala, Sweden), in a PyroMark MD instrument (Qiagen, Hilden, Germany) and PyroMark MD software Version 1.0.

## Results

### Profiling of fresh bone marrow aspirates with the AmpliSeq AML Research Panel

Starting with 40 ng of high-quality high molecular weight genomic DNA for each patient 6 fresh aspirate samples can be run in parallel on an Ion 318 Chip. The mean number of mapped reads per sample was around 1 million (Table 1) with more than 90% on-target and a mean depth per base approaching 5000 (more than 97% above 500 reads).

**Table 1:** Comparison of sequencing performance of the corresponding fresh and archival patient samples. Mapped reads, reads on target, mean coverage, and mean read length of the sequenced sample sets (each n = 10) of the aspirate DNA and three isolation methods of FFPE samples are shown. Additionally total reported variants are shown and the number of C>T/G>A variants below 10%.

Sample sets		Mean total reads	Mean reads on target	Mean coverage per base	Mean read length per amplicon [bp]	Total variants (n = )	C>T/G>A <10% variants (n = )
Aspirate	Mean Range	1,046,385 (690,053–1,334,001)	91.78% (87,67–95,06)	4556 (2832–5901)	137.7 (135–141)	261	19
Standard FFPE	Mean Range	680,528 (501,584–881,496)	86.89% (69,01–93,21)	2548 (1882–3643)	120.1 (107–125)	345	102
GeneRead kit	Mean Range	796,857 (433,502–1,463,615)	77.25% (58,45–85,60)	2592 (1455–4473)	114.6 (95–123)	282	39
Standard FFPE + UNG	Mean Range	853,043 (614,210–1,191,194)	94.46% (92,09–95,41)	3461 (2348–5072)	120.3 (113–125)	283	35

**Fig 2:**
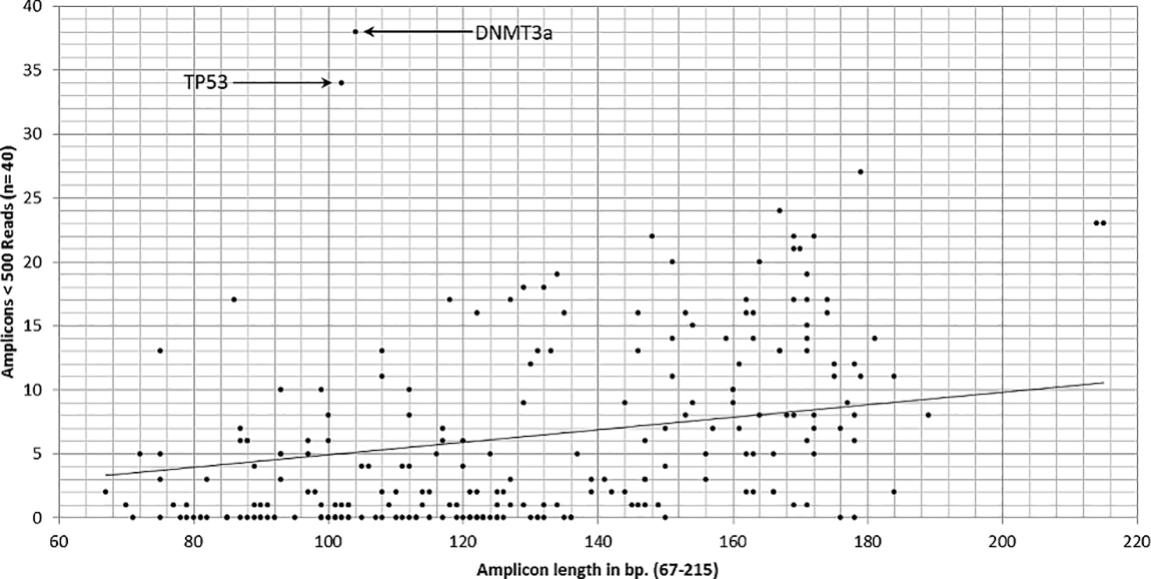
Low read depth versus amplicon length. Number of amplicons from the Ion AmpliSeq AML Research Panel sequenced with a read depth below 500x versus length of the amplicon. All samples from the training cohort in this study are shown (n = 40).

The uniformity of amplicon sequencing was very high. Nevertheless, individual amplicons performed much worse than the average. Namely, an amplicon covering part of exon 11 in the *TP53* gene (102 bp) and an amplicon covering part of exon 4 in the *DNMT3A* gene (104 bp) showed a very low representation in all sequenced samples, even in the high quality aspirate samples (see Fig 2). Altogether, 59 (2.5%) amplicons displayed a coverage below 500x in all 10 fresh aspirate samples. No correlation between amplicon length and obtained number of reads could be observed in aspirate samples, whereas a clear tendency of reduced mean amplicon coverage (MAC) in longer amplicons of FFPE samples can be observed, due to fragmentation of the input material (S1 and S2 Figs).

The MAC of all amplicons in the different sample sets including median values are shown in S3 Fig.

**Fig 3:**
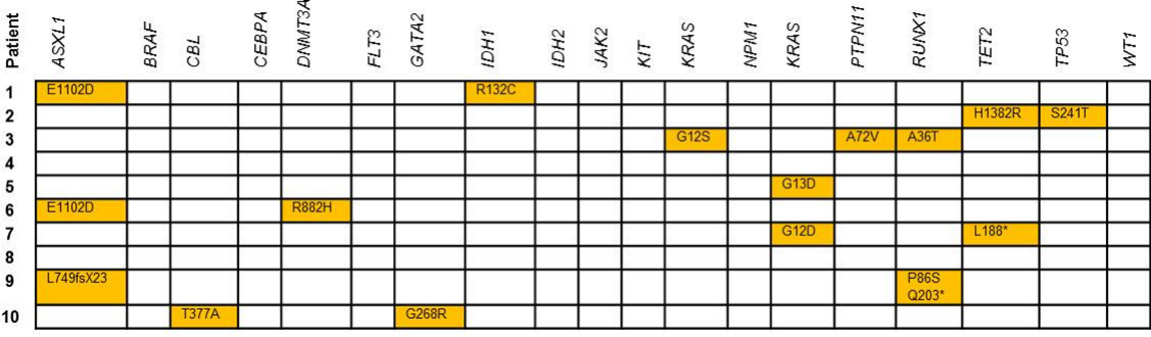
Overview of mutation calls in training cohort. In the 10 patient samples of the training cohort in total 17 mutations in 19 genes were filtered with Cartagenia Bench Lab NGS software (Version 3.1.2). Patient 9 shows two *RUNX1* mutations.

Altogether, 17 clinically relevant mutations were detected (Fig 3). Together with 25 well-annotated SNPs found in this sample set, these sequence variants were set as the gold standard for evaluation of the AmpliSeq AML panel in the corresponding formalin-fixed, decalcified and embedded bone marrow trephines.

### High concordance of fresh aspirate and corresponding trephines

Despite the fact that the overall yield and quality of reads obtained by sequencing DNA isolated by organic extraction from archival trephines was slightly reduced (see Table 1 for details) all known 17 diagnostically relevant mutations (distributed over 11 different genes) could be reliably identified in the series of corresponding trephines (formalin-fixed, decalcified, and paraffin-embedded). These trephines were taken from the same patient the very same day and match the freshly collected aspirate as close as possible.

In order to support these findings and to strengthen the technical validation of this new approach the coverage of 25 well-annotated SNPs found in the series of 10 fresh aspirates was analyzed in the corresponding trephines. The 25 SNPs could be identified in the aspirate samples with a mean coverage at the SNP of 4325 reads (range 1462–10202). In the corresponding trephine sample all 25 SNPs could be reliably verified with a mean coverage of 3242 reads (range 379–17553). The observed allele frequencies for all 25 SNPs were close to the expected value of 50%, with a mean frequency of 49.9% (range 47.4%-52.8%) in the aspirate samples and a mean frequency of 49.7% (range 40.9%-55.5%) in the corresponding trephines (see S2 Table for details). The allele frequencies, read depths and quality scores for the 17 diagnostically relevant mutations are presented in S3 Table.

### Reduction of formalin-induced artifacts by UNG pretreatment

The sequencing data of the trephine DNA isolated by organic extraction showed a very high number of C<T/G<A alterations with an allele frequency below 10% (Table 1, last column). These low frequency alterations are most probably formalin-fixation induced artifacts which may lead to false-positive mutation callings [12]. In addition, their sheer number makes the evaluation of the sequencing data more cumbersome and time-consuming. Therefore, protocols for reduction or even elimination of these alterations employing UNG glycosylase have been recently developed [13]. In order to test the reliability and robustness of this approach in combination with the AmpliSeq AML Research Panel a commercially available kit (GeneRead kit from Qiagen, Hilden, Germany) and a home-made UNG pre-treatment protocol were tested. The sequencing data were compared with the results obtained from the standard protocol for the trephines (exhaustive organic extraction) and the fresh aspirates.

**Fig 4:**
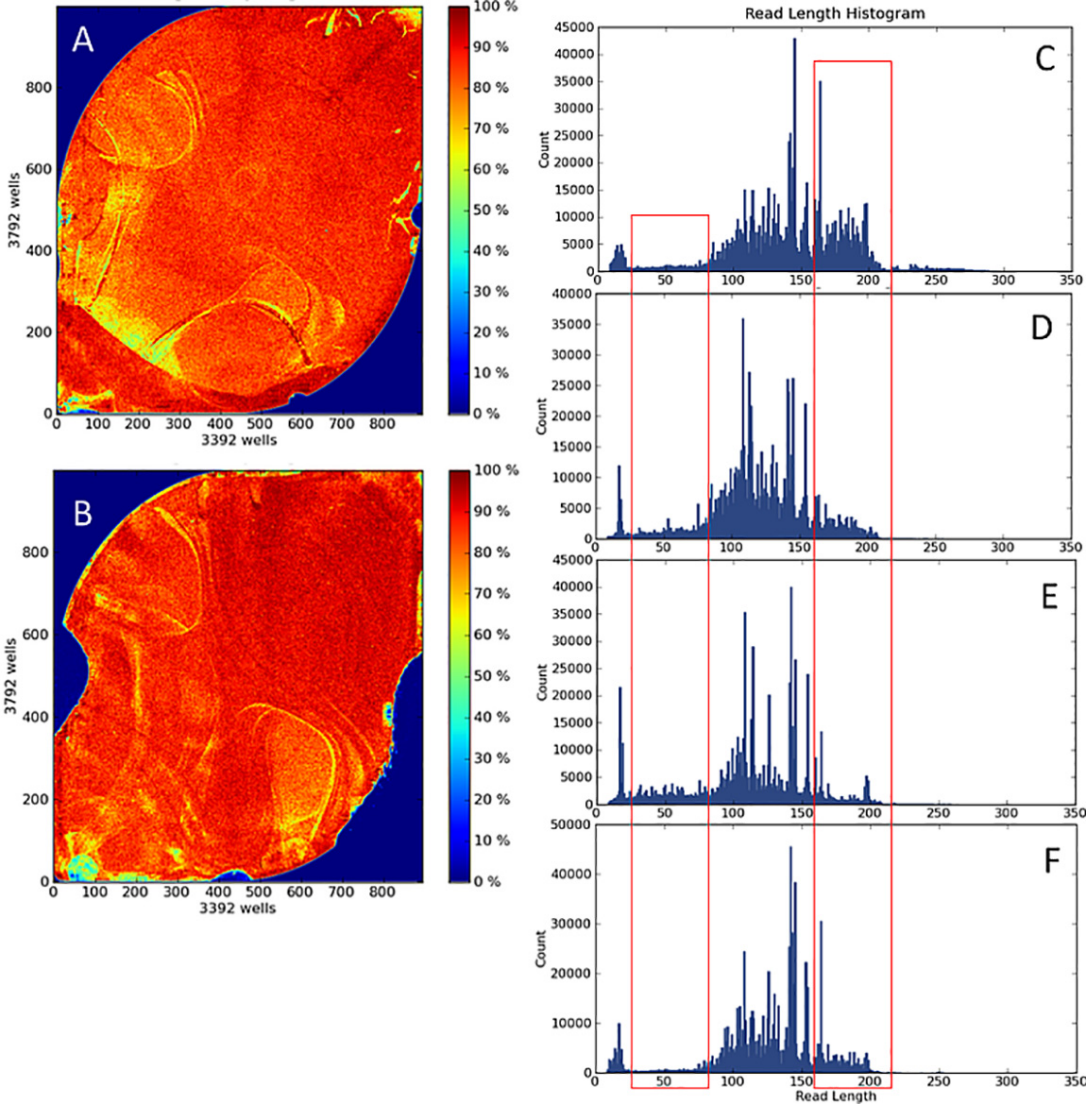
Technical run data from Torrent Server. (A) Loading intensity of 318 v2 chip with 6 aspirate samples. (B) Loading intensity of 318 v2 chip with 6 FFPE samples isolated with the standard protocol. Read length histogram represent selected samples from aspirate (C), standard protocol (D), GeneRead Kit (E), and standard protocol + UNG pre-treatment (F) sample sets.

Table 1 demonstrates that UNG pre-treatment clearly improves the overall quality metrics (number of reads, median sequence depth, respectively) and dramatically reduces the number of low-level (<10%) C<T/G<A alterations (by up to 65%). However, GeneRead Kit isolated samples produces less percentage of reads mapped on target compared with the standard protocol (77.3% vs. 86.9%). This leads to nearly identical mean coverage (2592 vs. 2548) despite more than 100,000 additional reads per sample in GeneRead Kit samples (796,857 vs. 680,528). Also mean read length is clearly reduced in GeneRead Kit samples (114.6) compared with both other FFPE isolation methods (120.1 using the standard protocol and 120.3 using the standard protocol+UNG pre-treatment, respectively). Thus, using the GeneRead Kit isolation method leads to shorter fragments of reduced quality for library preparation, which also would explain the reduced mean mapped reads on target. Fig 4 demonstrates that the overall loading of the sequencing chips is principally the same for DNA isolated from fresh aspirates or from archival trephines (compare panel A) and B) from Fig 4). The distribution of the read lengths, however, displays characteristic differences: nearly no short reads and larger amounts of longer reads in the fresh samples (Fig 4C)) whereas the archival trephines show reduced numbers of longer reads and an increase in short reads (Fig 4D–4F).

Following the standard procedure with additional UNG treatment for DNA isolation all 17 known mutations found in the fresh aspirate could be identified with reliable quality. Also the well-annotated 25 SNPs were identified with allele frequencies close to the expected value of 50% (mean frequency 49.7%, range 45.9%-56.3%).

**Fig 5:**
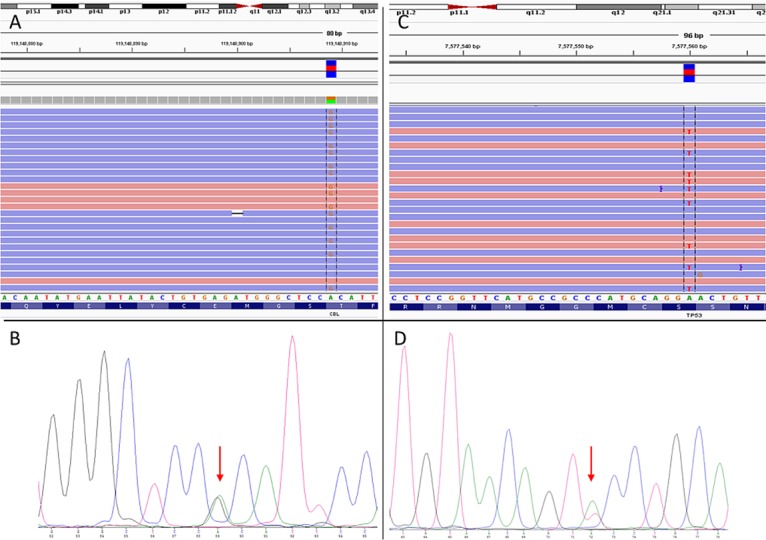
Verification of mutations from training cohort by Sanger sequencing. (A) Mapped reads of Patient 10 loaded in the IGV browser (Version 2.3.34). *CBL* p.T377A (c.1129G>A) variant with 50.6% variant frequency is shown. (B) Results from Sanger sequencing of *CBL* Exon 8. (C) Mapped reads of Patient 2 loaded in the IGV browser. *TP53* p.S241T (c.721T>A) with 24.7% variant frequency is shown (D) Results from Sanger sequencing of *TP53* Exon 7.

Using the GeneRead kit a single mutation could not be identified with sufficient quality (below a PHRED-scaled quality of 100). The data presented in Table 2 clearly show that the mutation is present in the data set. However, due to the overall reduced quality of the sequencing output from GeneRead Kit samples the number and quality of sequence reads covering this mutation did not meet all quality criteria. Fig 5 shows two single nucleotide variations from the training cohort which are successful verified by Sanger sequencing: a *CBL* p.T377A mutation with 50.6% frequency and a *TP53* p.S241T mutation with 24.7% allele frequency.

**Table 2:** Mutation profile of patient 9 (RAEB-1) from the training cohort. The quality (PHRED-scaled probability of incorrect calls) combines read depth and frequency of a given variant. The Ion Torrent software was adjusted to perform an analysis cut-off at 2000 reads.

Patient	Isolation method	Gene	Variant	Protein	Reads	Frequency [%]	Quality
9	Aspirate	*RUNX1*	c.256C>T	p.P86S	687	12.7	343.1
		*RUNX1*	c.607C>T	p.Q203X	3649	29.7	4410.9
		*ASXL1*	c.2246delT	p.L749fsX23	2862	44.3	8260.5
9	Standard FFPE	*RUNX1*	c.256C>T	p.P86S	920	12.6	450.9
		*RUNX1*	c.607C>T	p.Q203X	3546	24.6	3245.2
		*ASXL1*	c.2246delT	p.L749fsX23	1792	36.3	5671.3
9	GeneRead kit	*RUNX1*	c.256C>T	p.P86S	186	10.8	75.3
		*RUNX1*	c.607C>T	p.Q203X	2961	25.7	3490.0
		*ASXL1*	c.2246delT	p.L749fsX23	837	33.7	2239.3
9	Standard FFPE + UNG	*RUNX1*	c.256C>T	p.P86S	1604	12.2	725.2
		*RUNX1*	c.607C>T	p.Q203X	3807	26.5	3657.9
		*ASXL1*	c.2246delT	p.L749fsX23	2910	35.8	5888.6

### Performance of the AmpliSeq AML Research Panel under routine conditions

**Fig 6:**
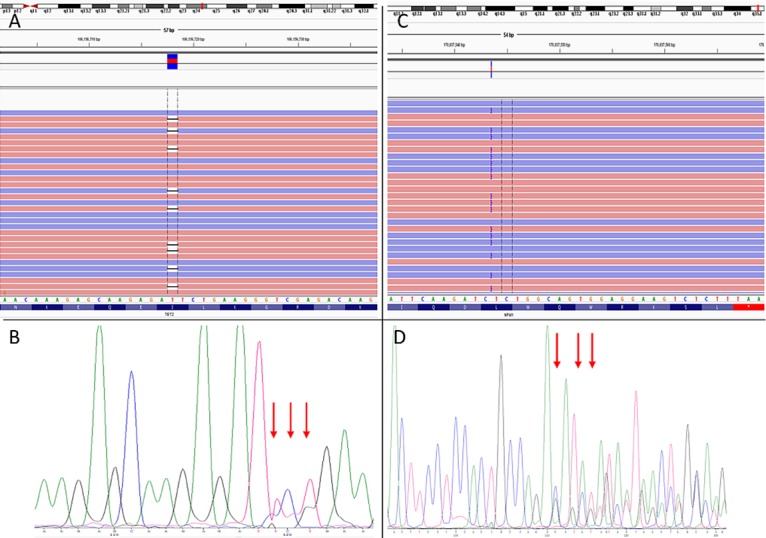
Verification of mutations from validation cohort by Sanger sequencing. (A) Mapped reads of a Patient with RCMD loaded in the IGV browser (Version 2.3.34). *TET2* p.L541X (c.1620delT) variant with 29.1% variant frequency in 592 reads is shown (B) Results from Sanger sequencing of *TET2* Exon 3. (C) Mapped reads of a Patient with AML loaded in the IGV browser. *NPM1* p.W288CfsX12 (c.860_863dupTCTG) mutation with 43.7% variant frequency is shown (D) Results from Sanger sequencing of *NPM1* Exon 12. Please note that Sanger sequencing shows the reverse sequence.

For validation of the AmpliSeq AML Research Panel under routine conditions we selected randomly 41 FFPE samples from the routine diagnostic work-up for semiconductor sequencing. The mean number of mapped reads was 716,788 (Range 272,434–1,093,052) and the median of average sequencing depth 2746 (Range 1097–4625). These values are very similar to those obtained within the training set. All known mutations (n = 43) could be verified by semiconductor sequencing. Additionally, 36 mutations in genes not covered by the routine diagnostic procedure could be identified. Table 3 gives an overview of the known and additionally found mutations in patient samples. Allele frequencies, read depths and methods used for validation are listed in S4 Table. From these 36 additional mutations selected were verified by Sanger sequencing. Fig 6 shows two complex mutations from the validation cohort: *TET2* p.L541X (c.1620delT) and *NPM1* p.W288CfsX12 (c.860_863dupTCTG). The *TET2* L541X mutation displayed an allele frequency of 29% in 592 reads (Quality 740.3). Despite this relatively low sequencing depth variant calling of this deletion was correct and could be verified by Sanger sequencing (Fig 6A and 6B). The *NPM1* W288CfsX12 mutation was sequenced with an allele-frequency of 44% in 3048 reads (Quality 8111.2) and could also be successfully verified (Fig 6C and 6D).

**Table 3:** Overview of verified known pathogenic mutations and additional detected variants in the validation cohort (n = 41). All variants are reported as mutations by Cartagenia Bench Lab NGS software (Version 3.1.2).

Verified known mutations (n = 43)		Additionally detected mutations (n = 36)			
*JAK2*	V617F	*ASXL1*	G710X	*TET2*	L541X
			Q910TfsX14		Q1030X
*NRAS*	G13D		L1393GfsX30		G1137D
			R1415X		L1151R
*NPM1*	W288CfsX12				R1261H
		*DNMT3A*	F751V		G1361C
*TP53*	V216M		R882C		H1381_H1382del
	P222L				V1417F
	P278T	*RUNX1*	N233LfsX50		V1718L
					H1904R
		*WT1*	D464N		

### Estimation of reagent costs and turn-around-time

In total we analyzed 81 DNA samples for this validation study of semiconductor sequencing with the Ion AmpliSeq AML Research Panel under routine conditions. For 12 patient samples Library preparation within two sequencing runs on 318 v2 chips we calculated reagent costs of 4,200 € (350 € per patient sample, Table 4). These costs are calculated with net list prices for Germany in March 2015. Taxes, salary, energy and maintenance costs or investments for equipment are not included.

**Table 4:** Estimation of the turn-around time and the costs for 12 samples processed with the AML panel and IonTorrent PGM semiconductor sequencer. Reagent costs are calculated from net list prices for Germany.

DNA isolation		Library preparation		Sequencing	
Duration:	2 days	Duration:	1 day	Duration:	7 hours (1 run)
Hands-on:	2 hours	Hands-on:	5 hours	Hands-on:	2 hours (1 run)
**Costs:**	**50 €**	**Costs:**	**2900 €**	**Costs:**	**1250 €**
● Isolation Kit/Chemicals		● Panel Primer		● Sequencing Kit	
● Quantification		● Library Kit		● 318 v2 Chips	
		● Barcodes			
		● Library Quantification			
		● Template preparation			

For DNA isolation from FFPE material the turn-around-time is 2 days, because of the required deparaffinisation of the sections and the prolonged incubation with Proteinase K overnight. For the exhaustive organic extraction also two days are necessary because of the precipitation overnight. During the first day one has an increased hands-on time of approx. 5 hours. Calculating one day for library preparation and one day for sequencing it is therefore possible to analyze 12 patient samples in four to five working days.

## Discussion

Comprehensive mutation profiling becomes more and more important in the routine diagnostic work up [14], especially of samples from patients with a suspected or confirmed hematological malignancy to make diagnosis, prognosis and therapy based on molecular markers more precisely [15]. Therefore, well established and validated primer sets for target enrichment of diagnostically relevant gene panels are required. The recently released AmpliSeq AML Research Panel from LifeTechnologies (now ThermoFisherScientific) represents a very promising contribution to this field. However, despite its long development time involving several research groups prior to the official launch and its release already in the middle of 2014 only very limited data about the performance of this panel are published. A single poster abstract authored by employees from LifeTechnologies/ThermoFisherScientific reporting on the analysis of 4 peripheral blood samples could be identified as of March 23^th^ 2015 (AACR proceedings 2014). Additionally, preliminary data are available through the website of the manufacturer.

Therefore, the results presented in this study are the first for the AmpliSeq AML Research Panel which are independent from the manufacturer. Basic performance characteristics observed by us, like number of reads obtained per sample, uniformity of amplification and reads mapped on target (see Table 1) demonstrate with very few exceptions a very high reproducibility and reliability of the target enrichment and subsequent sequencing even for DNA extracted from archival trephines. The latter fact is remarkable because the panel was developed and is validated so far only for the analysis of peripheral blood and bone marrow aspirate. The feasibility of using the AmpliSeq AML panel for FFPE trephines enables now the quick set up of mutational profiling in hematopathology for research studies and diagnostics.

The AmpliSeq AML Research Panel includes four large genes, which are impractical to be analyzed under routine conditions by Sanger-sequencing, because they would require numerous separate amplifications, PCR product purifications and sequencing reactions. The Panel covers the large exon 12 of *ASXL1*, all 23 exons of *DNMT3a*, exon 3 till 8 of *RUNX1*, and all 11 coding exons of *TET2*. We found at least one variant classified as pathogenic in these four genes in each patient of our training cohort: 3 in *ASXL1*, one in *DNMT3A*, 3 in *RUNX1*, and 2 variants in *TET2*, respectively (Fig 3). Furthermore we found 36 additional mutations in our validation cohort (patient samples n = 41), mostly in *ASXL1* and *TET2* (Table 3). This clearly demonstrates the usefulness of extending the diagnostic repertoire.

Due to the patent protection of the prognostically important detection of internal tandem duplications (ITD) in the exon14/exon15 region of the *FLT3* gene (patent hold by Takara.Inc, Japan) this genomic region was by purpose not covered by the amplicon design.

In many published studies using NGS techniques technical replicates have not been performed, even in validation studies introducing this technology [16–18]. We performed three independent DNA extractions from archival trephines, sequenced them in parallel and compared the obtained variant calls with each other and with those obtained from corresponding high-quality bone marrow aspirate DNA. These comparisons resulted in 98% concordance for all clinically relevant mutations (50 of 51) and 100% concordance for 25 well-annotated SNPs. In addition, the total number of variants identified in the four sets of DNA preparations was nearly identical, when C>T/G>A <10% variants where disregarded. In a single DNA preparation one *RUNX1* variant was sequenced correctly but did not fulfill the stringent quality criteria because of low coverage (see Table 2). Despite this we highly recommend a variant quality of at least 100 to minimize the risk of false-positive variant calls, especially when FFPE material is sequenced.

Our results concerning UNG pre-treatment confirm earlier reports [9,12,13] which could demonstrate a similar reduction in C:G>T:A single nucleotide changes by approximately 60%. These results complement each other because Do et al. 2013 [13] employed the TrueSeq Amplicon Cancer Panel from Illumina (San Diego, USA) which in contrast to the AmpliSeq technology from LifeTechnologies and sequenced the libraries using a MiSeq from Illumina. In our hands the GeneRead Kit did not work as good as our in-house protocol. This could be due to suboptimal purification (e.g., insufficient separation of smaller DNA fragments interfering with linker ligation and PCR during library construction) or the overall lower DNA yield.

With only 40 ng purified genomic DNA for semiconductor-based targeted resequencing it is possible to analyze 237 amplicons in 19 genes. This is much less starting material than other NGS applications require [19,20]. A couple of micrograms of DNA would be necessary to analyze a comparable number of genomic regions with Sanger sequencing. Usually those amounts cannot be obtained from FFPE material, which is also a strong argument for the introduction of NGS into the routine hematopathological diagnostic work-up.

We strongly recommend verifying whether mutations listed in the Ion Reporter variant list are represented in both strands of the amplicon containing the respective mutation. The independent amplification of both strands is a very important step in the quality control of the sequencing data in general.

Since Roche (Basel, Switzerland) announced end of last year to discontinue the production of the 454 sequencing platform (and will stop maintenance service in the near future) the study by Bernard et al. 2014 [20] about the applicability of NGS for the analysis of archival trephines lost its relevance for future developments in molecular hematopathology because the sequencing was performed on a Roche GS Junior bench-top sequencer. In addition, the scope of this validation study was quite limited: 3 genes (*TET2*, *CBL*, and *KRAS*) were analyzed in 4 corresponding fresh and archival bone marrow sample pairs. Subsequently, the authors analyzed these three genes in 26 bone marrow trephines.

In our previous study exploring the feasibility of NGS-based mutational profiling of bone marrow trephines [19] we employed reagents and a sequencer (Illumina HiSeq 2000) which are not compatible with the work-flow in the routine diagnostics. The target enrichment using long complementary RNA probes requires much more input DNA (hundreds of nanograms instead of 40 ng) which is very often simply not available. Sequencing on an Illumina HiSeq 2000 takes far too much time (10 days and more) for the routine work-up and reduces the scalability and flexibility of the work-flow substantially. In addition, the protocol used in that study requires much more hands-on time. All three factors prevent the implementation of this approach into daily routine practice and demonstrate the superior performance of the newly developed protocol.

In conclusion we could show that the recently AmpliSeq AML Research panel can also be used for the rapid, reliable and cost-effective mutational profiling of archival bone marrow trephines which now allows the implementation of NGS based sequence analyses into the histopathological and immunohistochemical examination of pathological conditions in the bone marrow. Also comprehensive retrospective analyses of large cohorts with well-annotated clinical data are now possible.

## Supporting Information

S1 Table Covered hot spot and coding regions from the 19 genes with relevance in AML which are included in the Ion AmpliSeq AML Research Panel. (DOCX)

S2 Table Comparison of 25 well-known SNPs which are identified in the aspirate samples and could be reliably confirmed in the corresponding trephines. (DOCX)

S3 Table Allele frequencies, read depths, and quality scores for the 17 diagnostically relevant mutations found in the aspirates and the corresponding trephines. (DOCX)

S4 Table Allele frequencies, read depths, and quality scores for the mutations found in 41 specimens from the validation cohort. The method used for independent validation is also indicated.
(DOCX)

S1 Fig Mean amplicon coverage of amplicons in 10 aspirate samples compared with amplicon length. Eleven amplicons which obtained more than 10,000 reads are not shown. The very weak positive correlation is regarded by us as not real (Spearman r = 0.211, p = 0.0014; linear regression r^2^ = 0.0414).
(TIF)

S2 Fig Mean amplicon coverage of amplicons in 10 FFPE bone marrow trephines (corresponding to the aspirates shown in S1 Fig) compared with amplicon length. Seven amplicons which obtained more than 10,000 reads are not shown. A clear negative correlation between amplicon length and read depth is discernible (Spearman r = -0.583, p < 0.0001; linear regression r^2^ = 0.205).
(TIF)

S3 Fig Mean amplicon coverage (MAC) for the four different DNA sample types analyzed in this study. The uniformity of the mean sequencing coverage for all 237 amplicons of the AML Panel within the four sample sets (each n = 10) is displayed. (A) MAC of the aspirate DNA samples (11 amplicons with mean coverage >10.000x are not included: 1x *NRAS*, 2x *DNMT3A*, 1x *GATA2*, 2x *TET2*, 4x *CEBP*α, and 1x *RUNX1*). (B) MAC of the standard protocol FFPE DNA samples (7 amplicons with a mean coverage >10.000x are not included: 1x amplicon *NRAS*, 2x *GATA2*, 2x *TET2*, 1x *CEBP*α, and 1x *RUNX1*). (C) MAC of GeneRead Kit isolated FFPE DNA samples (12 amplicons with a mean coverage >10.000x are not included: 1x amplicon *NRAS*, 3x *DNMT3A*, 2x *GATA2*, 2x *TET2*, 2x *CEBP*α, and 2x *RUNX1*). (D) MAC of standard protocol + UNG pre-treatment FFPE DNA samples (16 with a mean coverage >10.000x are not included: 1x amplicon *NRAS*, 3x *DNMT3A*, 3x *GATA2*, 1x *KIT*, 2x *TET2*, 1x *TP53*, 2x *CEBP*α, and 3x *RUNX1*). All amplicons representing one of the “large” genes (i.e., *DNMT3A*, *TET2*, and *ASXL1*) are highlighted by a colored box (grey: *DNMT3A*, red: *TET2*, yellow: *ASXL1*). The median amplicon coverage over all amplicons is indicated by a horizontal blue line. The lower read depth threshold for reliable evaluation of 500 reads per amplicon is indicated by a red horizontal line.
(TIF)
