# Supplementary material for: Comprehensive Molecular Profiling of Archival Bone Marrow Trephines Using a Commercially Available Leukemia Panel and Semiconductor-Based Targeted Resequencing
Source: PLoS One. 2015 Jul 29;10(7):e0133930. doi: 10.1371/journal.pone.0133930 (PMC4519100; doi:10.1371/journal.pone.0133930)
Supplement: S1 Table — (DOCX) [file pone.0133930.s001.docx]

Table S1 Covered hot spot and coding regions from the 19 genes with relevance in AML which are included in the Ion AmpliSeq™ AML Research Panel

| **Genes** | **Covered target region(s)** |
| --- | --- |
| *ASXL1* | exon 12 |
| *BRAF* | COSMIC mutation (COSM476) |
| *CBL* | exons 8, 9 |
| *CEBPA* | all coding exons |
| *DNMT3A* | all coding exons |
| *FLT3* | codons 676, 830-850 |
| *GATA2* | all coding exons |
| *IDH1* | exon 4 |
| *IDH2* | exon 4 |
| *JAK2* | exon 14 |
| *KIT* | exons 8, 10, 11, 17 |
| *KRAS* | exon 2, 3 |
| *NPM1* | exon 12 |
| *NRAS* | exons 2, 3 |
| *PTPN11* | exons 3, 7, 8, 13 |
| *RUNX1* | exons 3-8 |
| *TET2* | all coding exons |
| *TP53* | all coding exons |
| *WT1* | exons 7, 9 |

Total genomic area covered by the 237 amplicons: 30.4 kbp
