# Supplementary material for: Comprehensive Molecular Profiling of Archival Bone Marrow Trephines Using a Commercially Available Leukemia Panel and Semiconductor-Based Targeted Resequencing
Source: PLoS One. 2015 Jul 29;10(7):e0133930. doi: 10.1371/journal.pone.0133930 (PMC4519100; doi:10.1371/journal.pone.0133930)
Supplement: S2 Table — (DOCX) [file pone.0133930.s002.docx]

Table S2 Comparison of 25 well-known SNPs which are identified in the aspirate samples and could be reliably confirmed in the corresponding trephines

| **No. Variant** | **Sample** | **Location** | **Gene** | **Variant** | **Frequency** | **Reads** | **Quality** |
| --- | --- | --- | --- | --- | --- | --- | --- |
| 1 | 1 (Aspirate) | chr4:106,196,951 | TET2 | p.I1762V | 50.0 | 5902 | 10163.3 |
| 2 |  | chr17:7,579,472 | TP53 | p.P72R | 48.3 | 5320 | 9611.1 |
| 1 | 1 (GeneRead) | chr4:106,196,951 | TET2 | p.I1762V | 29.6 | 54 | 119.4 |
| 2 |  | chr17:7,579,472 | TP53 | p.P72R | 44.6 | 967 | 4112.4 |
| 1 | 1 (Standard) | chr4:106,196,951 | TET2 | p.I1762V | 40.9 | 613 | 2249.2 |
| 2 |  | chr17:7,579,472 | TP53 | p.P72R | 47.8 | 2781 | 9432.5 |
| 1 | 1 (Standard +UNG) | chr4:106,196,951 | TET2 | p.I1762V | 47.0 | 270 | 1223.0 |
| 2 |  | chr17:7,579,472 | TP53 | p.P72R | 49.1 | 1380 | 6812.9 |
| 3 | 2 (Aspirate) | chr20:31,024,274 | ASXL1 | p.S1253S | 50.0 | 5637 | 10148.3 |
| 3 | 2 (GeneRead) | chr20:31,024,274 | ASXL1 | p.S1253S | 46.4 | 1154 | 5171.9 |
| 3 | 2 (Standard) | chr20:31,024,274 | ASXL1 | p.S1253S | 45.2 | 1485 | 6427.2 |
| 3 | 2 (Standard+UNG) | chr20:31,024,274 | ASXL1 | p.S1253S | 50.2 | 900 | 4604.3 |
| 4 | 3 (Aspirate) | chr4:106,155,199 | TET2 | p.L34F | 51.0 | 4651 | 10443.1 |
| 5 |  | chr4:106,196,951 | TET2 | p.I1762V | 50.8 | 5824 | 10394.8 |
| 6 |  | chr17:7,579,472 | TP53 | p.P72R | 51.8 | 5150 | 10621.4 |
| 7 |  | chr20:31,024,274 | ASXL1 | p.S1253S | 47.6 | 7495 | 9393.3 |
| 4 | 3 (GeneRead) | chr4:106,155,199 | TET2 | p.L34F | 43.8 | 796 | 3284.5 |
| 5 |  | chr4:106,196,951 | TET2 | p.I1762V | 41.4 | 239 | 904.1 |
| 6 |  | chr17:7,579,472 | TP53 | p.P72R | 44.5 | 1527 | 6463.6 |
| 7 |  | chr20:31,024,274 | ASXL1 | p.S1253S | 49.5 | 564 | 2784.2 |
| 4 | 3 (Standard) | chr4:106,155,199 | TET2 | p.L34F | 52.1 | 2225 | 10795.7 |
| 5 |  | chr4:106,196,951 | TET2 | p.I1762V | 45.9 | 1331 | 5898.1 |
| 6 |  | chr17:7,579,472 | TP53 | p.P72R | 50.3 | 4744 | 10121.6 |
| 7 |  | chr20:31,024,274 | ASXL1 | p.S1253S | 54.8 | 1345 | 7895.0 |
| 4 | 3 (Standard+UNG) | chr4:106,155,199 | TET2 | p.L34F | 47.1 | 873 | 4013.7 |
| 5 |  | chr4:106,196,951 | TET2 | p.I1762V | 53.5 | 408 | 2271.4 |
| 6 |  | chr17:7,579,472 | TP53 | p.P72R | 52.0 | 1387 | 7509.4 |
| 7 |  | chr20:31,024,274 | ASXL1 | p.S1253S | 46.5 | 576 | 2586.2 |
| 8 | 4 (Aspirate) | chr3:128200072 | GATA2 | p.A411A | 50.2 | 2597 | 10206.3 |
| 9 |  | chr17:7,579,472 | TP53 | p.P72R | 50.1 | 5313 | 9959.9 |
| 8 | 4 (GeneRead) | chr3:128200072 | GATA2 | p.A411A | 51.3 | 1908 | 10022.7 |
| 9 |  | chr17:7,579,472 | TP53 | p.P72R | 48.6 | 2419 | 9727.2 |
| 8 | 4 (Standard) | chr3:128200072 | GATA2 | p.A411A | 53.0 | 2536 | 11131.1 |
| 9 |  | chr17:7,579,472 | TP53 | p.P72R | 53.3 | 4240 | 10976.7 |
| 8 | 4 (Standard +UNG) | chr3:128200072 | GATA2 | p.A411A | 48.2 | 2153 | 9576.8 |
| 9 |  | chr17:7,579,472 | TP53 | p.P72R | 47.2 | 2332 | 9280.1 |
| 10 | 5 (Aspirate) | chr4:106,156,187 | TET2 | p.P363L | 49.3 | 1462 | 7215.7 |
| 11 |  | chr4:106,196,951 | TET2 | p.I1762V | 49.1 | 1822 | 8966.6 |
| 12 |  | chr20:31,024,274 | ASXL1 | p.S1253S | 49.6 | 2126 | 10018.2 |
| 10 | 5 (GeneRead) | chr4:106,156,187 | TET2 | p.P363L | 50.8 | 1134 | 5920.9 |
| 11 |  | chr4:106,196,951 | TET2 | p.I1762V | 51.5 | 235 | 1255.2 |
| 12 |  | chr20:31,024,274 | ASXL1 | p.S1253S | 49.5 | 586 | 2923.2 |
| 10 | 5 (Standard) | chr4:106,156,187 | TET2 | p.P363L | 55.5 | 573 | 3399.5 |
| 11 |  | chr4:106,196,951 | TET2 | p.I1762V | 54.6 | 388 | 2260.4 |
| 12 |  | chr20:31,024,274 | ASXL1 | p.S1253S | 44.9 | 379 | 1611.4 |
| 10 | 5 (Standard+UNG) | chr4:106,156,187 | TET2 | p.P363L | 45.9 | 864 | 3812.9 |
| 11 |  | chr4:106,196,951 | TET2 | p.I1762V | 50.7 | 399 | 2094.7 |
| 12 |  | chr20:31,024,274 | ASXL1 | p.S1253S | 56.3 | 573 | 3473.3 |
| 13 | 6 (Aspirate) | chr3:128,205,860 | GATA2 | p.P5P | 49.6 | 7560 | 10017.2 |
| 14 |  | chr17:7,579,472 | TP53 | p.P72R | 47.4 | 1978 | 9214.4 |
| 13 | 6 (GeneRead) | chr3:128,205,860 | GATA2 | p.P5P | 49.7 | 12667 | 10034.3 |
| 14 |  | chr17:7,579,472 | TP53 | p.P72R | 49.0 | 496 | 2403.7 |
| 13 | 6 (Standard) | chr3:128,205,860 | GATA2 | p.P5P | 48.0 | 14237 | 9464.6 |
| 14 |  | chr17:7,579,472 | TP53 | p.P72R | 46.4 | 1489 | 6720.1 |
| 13 | 6 (Standard +UNG) | chr3:128,205,860 | GATA2 | p.P5P | 47.8 | 18575 | 9461.6 |
| 14 |  | chr17:7,579,472 | TP53 | p.P72R | 46.4 | 2298 | 9033.2 |
| 15 | 7 (Aspirate) | chr3:128,205,860 | GATA2 | p.P5P | 51.1 | 6011 | 10470.6 |
| 15 | 7 (GeneRead) | chr3:128,205,860 | GATA2 | p.P5P | 49.2 | 10868 | 9883.6 |
| 15 | 7 (Standard) | chr3:128,205,860 | GATA2 | p.P5P | 48.9 | 4829 | 9783.3 |
| 15 | 7 (Standard+UNG) | chr3:128,205,860 | GATA2 | p.P5P | 49.1 | 15193 | 9883.2 |
| 16 | 8 (Aspirate) | chr4:106,196,951 | TET2 | p.I1762V | 50.5 | 3237 | 10328.5 |
| 17 |  | chr3:128,204,951 | GATA2 | p.A164T | 52.2 | 2668 | 10837.6 |
| 18 |  | chr2:25,469,502 | DNMT3A | p.L421L | 47.8 | 3521 | 9949.0 |
| 16 | 8 (GeneRead) | chr4:106,196,951 | TET2 | p.I1762V | 47.5 | 366 | 1689.6 |
| 17 |  | chr3:128,204,951 | GATA2 | p.A164T | 59.6 | 411 | 2702.4 |
| 18 |  | chr2:25,469,502 | DNMT3A | p.L421L | 51.3 | 238 | 1253.7 |
| 16 | 8 (Standard) | chr4:106,196,951 | TET2 | p.I1762V | 49.7 | 481 | 2406.9 |
| 17 |  | chr3:128,204,951 | GATA2 | p.A164T | 47.6 | 2811 | 9399.5 |
| 18 |  | chr2:25,469,502 | DNMT3A | p.L421L | 47.5 | 1568 | 7323.1 |
| 16 | 8 (Standard+UNG) | chr4:106,196,951 | TET2 | p.I1762V | 50.5 | 1119 | 5755.4 |
| 17 |  | chr3:128,204,951 | GATA2 | p.A164T | 53.7 | 2186 | 11361.5 |
| 18 |  | chr2:25,469,502 | DNMT3A | p.L421L | 53.6 | 2084 | 11320.4 |
| 19 | 9 (Aspirate) | chr4:106,196,951 | TET2 | p.I1762V | 47.6 | 3164 | 9415.1 |
| 20 |  | chr3:128,204,951 | GATA2 | p.A164T | 51.4 | 2586 | 10551.1 |
| 21 |  | chr3:128,205,860 | GATA2 | p.P5P | 48.4 | 10202 | 9589.4 |
| 19 | 9 (GeneRead) | chr4:106,196,951 | TET2 | p.I1762V | 44.7 | 179 | 751.0 |
| 20 |  | chr3:128,204,951 | GATA2 | p.A164T | 49.5 | 515 | 2534.5 |
| 21 |  | chr3:128,205,860 | GATA2 | p.P5P | 51.1 | 11683 | 10465.2 |
| 19 | 9 (Standard) | chr4:106,196,951 | TET2 | p.I1762V | 45.6 | 754 | 3283.4 |
| 20 |  | chr3:128,204,951 | GATA2 | p.A164T | 54.6 | 1638 | 9546.3 |
| 21 |  | chr3:128,205,860 | GATA2 | p.P5P | 49.8 | 17553 | 10000.7 |
| 19 | 9 (Standard+UNG) | chr4:106,196,951 | TET2 | p.I1762V | 50.0 | 1209 | 6863.5 |
| 20 |  | chr3:128,204,951 | GATA2 | p.A164T | 53.6 | 2473 | 11323.0 |
| 21 |  | chr3:128,205,860 | GATA2 | p.P5P | 49.5 | 18386 | 9942.2 |
| 22 | 10 (Aspirate) | chr4:106,156,163 | TET2 | p.G355D | 52.8 | 2675 | 11056.9 |
| 23 |  | chr4:106,196,951 | TET2 | p.I1762V | 50.9 | 2588 | 10460.2 |
| 24 |  | chr3:128,205,860 | GATA2 | p.P5P | 51.0 | 10062 | 10444.6 |
| 25 |  | chr17:7,579,472 | TP53 | p.P72R | 48.1 | 2062 | 9570.1 |
| 22 | 10 (GeneRead) | chr4:106,156,163 | TET2 | p.G355D | 48.3 | 1045 | 5040.6 |
| 23 |  | chr4:106,196,951 | TET2 | p.I1762V | 51.0 | 333 | 1752.8 |
| 24 |  | chr3:128,205,860 | GATA2 | p.P5P | 49.9 | 8727 | 10061.1 |
| 25 |  | chr17:7,579,472 | TP53 | p.P72R | 48.4 | 749 | 3579.1 |
| 22 | 10 (Standard) | chr4:106,156,163 | TET2 | p.G355D | 50.4 | 1019 | 5215.9 |
| 23 |  | chr4:106,196,951 | TET2 | p.I1762V | 49.1 | 615 | 3004.9 |
| 24 |  | chr3:128,205,860 | GATA2 | p.P5P | 49.9 | 10060 | 10118.6 |
| 25 |  | chr17:7,579,472 | TP53 | p.P72R | 52.3 | 1178 | 6376.8 |
| 22 | 10 (Standard+UNG) | chr4:106,156,163 | TET2 | p.G355D | 46.6 | 2552 | 9088.6 |
| 23 |  | chr4:106,196,951 | TET2 | p.I1762V | 48.3 | 1229 | 5890.6 |
| 24 |  | chr3:128,205,860 | GATA2 | p.P5P | 51.2 | 26571 | 10502.0 |
| 25 |  | chr17:7,579,472 | TP53 | p.P72R | 49.0 | 2930 | 9829.9 |
