# Supplementary material for: Comprehensive Molecular Profiling of Archival Bone Marrow Trephines Using a Commercially Available Leukemia Panel and Semiconductor-Based Targeted Resequencing
Source: PLoS One. 2015 Jul 29;10(7):e0133930. doi: 10.1371/journal.pone.0133930 (PMC4519100; doi:10.1371/journal.pone.0133930)
Supplement: S3 Table — (DOCX) [file pone.0133930.s003.docx]

Table S3 Overview of detected pathogenic mutations which are identified in the aspirate samples and could be reliably confirmed in the corresponding trephines.

In the first column in the first row for each patient the blast count determined in the bone marrow trephine is indicated.

| **Patient** | **Location** | **Gene** | **Sequence** | **Protein** | **Frequency** | **Reads** | **Quality** |
| --- | --- | --- | --- | --- | --- | --- | --- |
| 1 (Aspirate) 10% | chr20:31023821 | ASXL1 | c.3306G>T | p.E1102D | 46.80% | 4501 | 8883.3 |
|  | chr2:209113113 | IDH1 | c.394C>T | p.R132C | 47.30% | 3683 | 9308 |
| 1 (Standard) | chr20:31023821 | ASXL1 | c.3306G>T | p.E1102D | 51.30% | 567 | 2881.4 |
|  | chr2:209113113 | IDH1 | c.394C>T | p.R132C | 46.00% | 421 | 1858.1 |
| 1 (GeneRead) | chr20:31023821 | ASXL1 | c.3306G>T | p.E1102D | 47.10% | 67 | 292.8 |
|  | chr2:209113113 | IDH1 | c.394C>T | p.R132C | 36.40% | 176 | 532.4 |
| 1 (Standard + UNG) | chr20:31023821 | ASXL1 | c.3306G>T | p.E1102D | 49.20% | 411 | 1983.9 |
|  | chr2:209113113 | IDH1 | c.394C>T | p.R132C | 46.90% | 371 | 1672.2 |
| 2 (Aspirate) <5% | chr4:106190867 | TET2 | c.4145A>G | p.H1382R | 33.10% | 2798 | 5269.6 |
|  | chr17:7577560 | TP53 | C.721T>A | p.S241T | 24.70% | 5117 | 3265 |
| 2 (Standard) | chr4:106190867 | TET2 | c.4145A>G | p.H1382R | 43.00% | 2110 | 8000.8 |
|  | chr17:7577560 | TP53 | C.721T>A | p.S241T | 27.10% | 4477 | 3791.7 |
| 2 (GeneRead) | chr4:106190867 | TET2 | c.4145A>G | p.H1382R | 42.30% | 60147 | 7786.3 |
|  | chr17:7577560 | TP53 | C.721T>A | p.S241T | 28.60% | 2387 | 4156.4 |
| 2 (Standard + UNG) | chr4:106190867 | TET2 | c.4145A>G | p.H1382R | 41.80% | 18496 | 7650.1 |
|  | chr17:7577560 | TP53 | C.721T>A | p.S241T | 27.50% | 1550 | 3007.1 |
| 3 (Aspirate) 10% | chr12:112888199 | PTPN11 | c.215C>T | p.A72V | 40.60% | 7908 | 7291.2 |
|  | chr12:25398285 | KRAS | c.34G>A | p.G12S | 10.40% | 2530 | 1369.6 |
|  | chr21:36259304 | RUNX1 | c106G>A | p.A36T | 24.00% | 2069 | 2689.1 |
| 3 (Standard) | chr12:112888199 | PTPN11 | c.215C>T | p.A72V | 44.80% | 1800 | 7729.7 |
|  | chr12:25398285 | KRAS | c.34G>A | p.G12S | 11.20% | 808 | 404.3 |
|  | chr21:36259304 | RUNX1 | c106G>A | p.A36T | 22.80% | 1025 | 1596.3 |
| 3 (GeneRead) | chr12:112888199 | PTPN11 | c.215C>T | p.A72V | 33.40% | 436 | 1173.7 |
|  | chr12:25398285 | KRAS | c.34G>A | p.G12S | 13.80% | 569 | 327.6 |
|  | chr21:36259304 | RUNX1 | c106G>A | p.A36T | 21.30% | 971 | 1425.6 |
| 3 (Standard + UNG) | chr12:112888199 | PTPN11 | c.215C>T | p.A72V | 40.70% | 520 | 1895.9 |
|  | chr12:25398285 | KRAS | c.34G>A | p.G12S | 11.80% | 608 | 311.8 |
|  | chr21:36259304 | RUNX1 | c106G>A | p.A36T | 24.40% | 659 | 978.9 |
| 4 (Aspirate) 25% | No pathogenic variants detected | | | | | | |
| 4 (Standard) | No pathogenic variants detected | | | | | | |
| 4 (GeneRead) | No pathogenic variants detected | | | | | | |
| 4 (Standard + UNG) | No pathogenic variants detected | | | | | | |
| 5 (Aspirate) <5% | chr1:115258744 | NRAS | c.38G>A | p.G13D | 14.50% | 2266 | 1253.8 |
| 5 (Standard) | chr1:115258744 | NRAS | c.38G>A | p.G13D | 11.70% | 894 | 379.7 |
| 5 (GeneRead) | chr1:115258744 | NRAS | c.38G>A | p.G13D | 9.70% | 344 | 107 |
| 5 (Standard + UNG) | chr1:115258744 | NRAS | c.38G>A | p.G13D | 13.50% | 558 | 311.6 |
| 6 (Aspirate) 5 – 6% | chr2:25457242 | DNMT3a | c.2645G>A | p.R882H | 43.10% | 5871 | 7998.1 |
|  | chr20:31023821 | ASXL1 | c.3306G>T | p.E1102D | 46.60% | 2554 | 8931.1 |
| 6 (Standard) | chr2:25457242 | DNMT3a | c.2645G>A | p.R882H | 38.30% | 1379 | 4596.4 |
|  | chr20:31023821 | ASXL1 | c.3306G>T | p.E1102D | 50.50% | 732 | 3652.2 |
| 6 (GeneRead) | chr2:25457242 | DNMT3a | c.2645G>A | p.R882H | 47.50% | 4066 | 9352 |
|  | chr20:31023821 | ASXL1 | c.3306G>T | p.E1102D | 15.60% | 376 | 281.2 |
| 6 (Standard + UNG) | chr2:25457242 | DNMT3a | c.2645G>A | p.R882H | 42.00% | 1557 | 5979 |
|  | chr20:31023821 | ASXL1 | c.3306G>T | p.E1102D | 45.20% | 888 | 3721.4 |
| 7 (Aspirate) 10% | chr1:115258747 | NRAS | c.35G>A | p.G12D | 46.40% | 2903 | 9027.3 |
|  | chr4:106155661 | TET2 | c.562A>T | p.L188* | 93.10% | 2189 | 27621.4 |
| 7 (Standard) | chr1:115258747 | NRAS | c.35G>A | p.G12D | 40.00% | 1634 | 5826.7 |
|  | chr4:106155661 | TET2 | c.562A>T | p.L188* | 84.80% | 469 | 5541.8 |
| 7 (GeneRead) | chr1:115258747 | NRAS | c.35G>A | p.G12D | 39.70% | 594 | 2106.8 |
|  | chr4:106155661 | TET2 | c.562A>T | p.L188* | 79.80% | 287 | 3039.5 |
| 7 (Standard + UNG) | chr1:115258747 | NRAS | c.35G>A | p.G12D | 37.70% | 1186 | 3853.3 |
|  | chr4:106155661 | TET2 | c.562A>T | p.L188* | 83.10% | 731 | 8309.1 |
| 8 (Aspirate) 20% | No pathogenic variants detected | | | | | | |
| 8 (Standard) | No pathogenic variants detected | | | | | | |
| 8 (GeneRead) | No pathogenic variants detected | | | | | | |
| 8 (Standard + UNG) | No pathogenic variants detected | | | | | | |
| 9 (Aspirate) 6% | chr21:36259154 | RUNX1 | c.256C>T | p.P86S | 12.70% | 687 | 343.1 |
|  | chr21:36206824 | RUNX1 | c.607C>T | p.Q203* | 29.70% | 3649 | 4410.9 |
|  | chr20:31022761 | ASXL1 | c.2246delT | p.L749fs*23 | 44.30% | 2862 | 8260.5 |
| 9 (Standard) | chr21:36259154 | RUNX1 | c.256C>T | p.P86S | 12.60% | 920 | 450.9 |
|  | chr21:36206824 | RUNX1 | c.607C>T | p.Q203* | 24.60% | 3546 | 3245.2 |
|  | chr20:31022761 | ASXL1 | c.2246delT | p.L749fs*23 | 36.30% | 1792 | 5671.3 |
| 9 (GeneRead) | chr21:36259154 | RUNX1 | c.256C>T | p.P86S | 10.80% | 186 | 75.3 |
|  | chr21:36206824 | RUNX1 | c.607C>T | p.Q203* | 25.70% | 2961 | 3490 |
|  | chr20:31022761 | ASXL1 | c.2246delT | p.L749fs*23 | 33.70% | 837 | 2239.3 |
| 9 (Standard + UNG) | chr21:36259154 | RUNX1 | c.256C>T | p.P86S | 12.20% | 1604 | 725.2 |
|  | chr21:36206824 | RUNX1 | c.607C>T | p.Q203* | 26.50% | 3807 | 3657.9 |
|  | chr20:31022761 | ASXL1 | c.2246delT | p.L749fs*23 | 35.80% | 2910 | 5888.6 |
| 10 (Aspirate) 4-5% | chr3:128204639 | GATA2 | c.802G>A | p.G268R | 49.80% | 2260 | 10062.3 |
|  | chr11:119148909 | CBL | c.1129A>G | p.T377A | 50.60% | 1087 | 5606.1 |
| 10 (Standard) | chr3:128204639 | GATA2 | c.802G>A | p.G268R | 53.20% | 1345 | 7497.7 |
|  | chr11:119148909 | CBL | c.1129A>G | p.T377A | 51.40% | 783 | 4149.1 |
| 10 (GeneRead) | chr3:128204639 | GATA2 | c.802G>A | p.G268R | 51.10% | 1719 | 8871.7 |
|  | chr11:119148909 | CBL | c.1129A>G | p.T377A | 53.00% | 83 | 450.6 |
| 10 (Standard + UNG) | chr3:128204639 | GATA2 | c.802G>A | p.G268R | 52.20% | 3968 | 10835 |
|  | chr11:119148909 | CBL | c.1129A>G | p.T377A | 55.30% | 673 | 3972.3 |
