# Supplementary material for: Comprehensive Molecular Profiling of Archival Bone Marrow Trephines Using a Commercially Available Leukemia Panel and Semiconductor-Based Targeted Resequencing
Source: PLoS One. 2015 Jul 29;10(7):e0133930. doi: 10.1371/journal.pone.0133930 (PMC4519100; doi:10.1371/journal.pone.0133930)
Supplement: S4 Table — The method used for independent validation is also indicated. (DOCX) [file pone.0133930.s004.docx]

Table S4 Overview of detected pathogenic Mutations in 41 samples of the validation cohort, which are identified with the AML research panel. If stated, the mutations are initially detected with Pyro- or Sangersequencing and successfully verified by semiconductor sequencing.

| **Sample** | **Location** | **Gene** | **Sequence** | **Protein** | **Frequency** | **Reads** | **Quality** | **Initially detected** |
| --- | --- | --- | --- | --- | --- | --- | --- | --- |
| 1 | chr4:106158187 | TET2 | c.3088C>T | p.Q1030* | 28.30% | 92 | 194.7 | n.d. |
|  | chr4:106162538 | TET2 | c.3452T>G | p.L1151R | 44.40% | 45 | 194.9 | n.d. |
|  | chr9:5073770 | JAK2 | c.1849G>T | p.V617F | 57.40% | 55 | 307 | Pyro |
|  | chr17:7578184 | TP53 | c.665C>T | p.P222L | 50.00% | 77 | 380.8 | Sanger |
| 2 | chr4:106158187 | TET2 | c.3088C>T | p.Q1030* | 40.30% | 1459 | 5246 | n.d. |
|  | chr4:106162538 | TET2 | c.3452T>G | p.L1151R | 44.80% | 958 | 4069.2 | n.d. |
|  | chr9:5073770 | JAK2 | c.1849G>T | p.V617F | 52.20% | 306 | 1716.2 | Pyro |
|  | chr17:7578184 | TP53 | c.665C>T | p.P222L | 49.60% | 2110 | 10007.7 | Sanger |
| 3 | chr4:106158187 | TET2 | c.3088C>T | p.Q1030* | 31.40% | 86 | 210.3 | n.d. |
|  | chr4:106162538 | TET2 | c.3452T>G | p.L1151R | 38.50% | 52 | 169.2 | n.d. |
|  | chr9:5073770 | JAK2 | c.1849G>T | p.V617F | 84.20% | 57 | 597.4 | Pyro |
|  | chr17:7578184 | TP53 | c.665C>T | p.P222L | 46.80% | 47 | 222.9 | Sanger |
| 4 | chr4:106158187 | TET2 | c.3088C>T | p.Q1030* | 44.90% | 1836 | 7875.5 | n.d. |
|  | chr4:106162538 | TET2 | c.3452T>G | p.L1151R | 50.70% | 1108 | 5710.4 | n.d. |
|  | chr9:5073770 | JAK2 | c.1849G>T | p.V617F | 88.40% | 442 | 5700.7 | Pyro |
|  | chr17:7578184 | TP53 | c.665C>T | p.P222L | 54.20% | 2344 | 11503.9 | Sanger |
| 5 | chr9:5073770 | JAK2 | c.1849G>T | p.V617F | 68.40% | 57 | 424.7 | Pyro |
|  | chr20:31024689 | ASXL1 | c.4176_4177delCC | p.L1393Gfs*30 | 19.40% | 102 | 62.8 | n.d. |
| 6 | chr4:106164914 | TET2 | c.3782G>A | p.R1261H | 21.30% | 94 | 128.2 | n.d. |
|  | chr9:5073770 | JAK2 | c.1849G>T | p.V617F | 19.20% | 1206 | 859.3 | Pyro |
| 7 | No pathogenic variants detected | | | | | | | |
| 8 | chr9:5073770 | JAK2 | c.1849G>T | p.V617F | 42.30% | 71 | 261.6 | Pyro |
|  | chr20:31024689 | ASXL1 | c.4176_4177delCC | p.L1393Gfs*30 | 23.10% | 109 | 93.6 | n.d. |
| 9 | chr4:106196819 | TET2 | c.5152G>T | p.V1718L | 50.90% | 467 | 2415.9 | n.d. |
| 10 | chr4:106193787 | TET2 | c.4249G>T | p.V1417F | 41.30% | 1005 | 4279 | n.d. |
|  | chr4:106196819 | TET2 | c.5152G>T | p.V1718L | 49.50% | 1445 | 7259.5 | n.d. |
|  | chr9:5073770 | JAK2 | c.1849G>T | p.V617F | 75.80% | 967 | 9629.6 | Pyro |
|  | chr20:31023240 | ASXL1 | c.2727dupA | p.Q910Tfs*14 | 13.00% | 1815 | 227 | n.d. |
| 11 | chr9:5073770 | JAK2 | c.1849G>T | p.V617F | 29.00% | 339 | 725.8 | Pyro |
|  | chr20:31024758 | ASXL1 | c.4243C>T | p.R1415* | 14.40% | 1400 | 884.8 | n.d. |
| 12 | chr9:5073770 | JAK2 | c.1849G>T | p.V617F | 75.40% | 581 | 5622.6 | Pyro |
|  | chr20:31023821 | ASXL1 | c.3306G>T | p.E1102D | 41.40% | 772 | 2846.3 | n.d. |
| 13 | chr9:5073770 | JAK2 | c.1849G>T | p.V617F | 61.50% | 616 | 4281.4 | Pyro |
|  | chr20:31024488 | ASXL1 | c.3973C>T | p.L1325F | 58.50% | 479 | 3108.7 | n.d. |
| 14 | No pathogenic variants detected | | | | | | | |
| 15 | chr9:5073770 | JAK2 | c.1849G>T | p.V617F | 58.50% | 225 | 1470.1 | Pyro |
| 16 | chr9:5073770 | JAK2 | c.1849G>T | p.V617F | 91.50% | 185 | 2531.2 | Pyro |
| 17 | chr9:5073770 | JAK2 | c.1849G>T | p.V617F | 46.60% | 588 | 2685.6 | Pyro |
| 18 | No pathogenic variants detected | | | | | | | |
| 19 | chr9:5073770 | JAK2 | c.1849G>T | p.V617F | 69.20% | 208 | 1700.8 | n.d. |
|  | chr4:106158509 | TET2 | c.3410G>A | p.G1137D | 30.90% | 956 | 2237.1 | n.d. |
|  | chr4:106190803 | TET2 | c.4081G>T | p.G1361C | 47.00% | 37960 | 9227 | n.d. |
|  | chr4:106197378 | TET2 | c.5711A>G | p.H1904R | 6.00% | 2028 | 180 | n.d. |
|  | chr17:7577106 | TP53 | c.832C>A | p.P278T | 5.60% | 7436 | 141.1 | Sanger |
| 20 | chr9:5073770 | JAK2 | c.1849G>T | p.V617F | 62.40% | 252 | 1803.3 | Pyro |
| 21 | chr9:5073770 | JAK2 | c.1849G>T | p.V617F | 84.80% | 428 | 5252.6 | Pyro |
| 22 | chr9:5073770 | JAK2 | c.1849G>T | p.V617F | 70.70% | 540 | 4952 | Pyro |
| 23 | chr9:5073770 | JAK2 | c.1849G>T | p.V617F | 81.00% | 1134 | 12913.9 | Pyro |
| 24 | chr9:5073770 | JAK2 | c.1849G>T | p.V617F | 76.80% | 742 | 7633.1 | Pyro |
| 25 | chr9:5073770 | JAK2 | c.1849G>T | p.V617F | 76.00% | 2388 | 19668.4 | Pyro |
| 26 | No pathogenic variants detected | | | | | | | |
| 27 | chr9:5073770 | JAK2 | c.1849G>T | p.V617F | 82.30% | 432 | 4859.1 | Pyro |
|  | chr20:31023821 | ASXL1 | c.3306G>T | p.E1102D | 47.80% | 2537 | 9420.1 | n.d. |
| 28 | chr9:5073770 | JAK2 | c.1849G>T | p.V617F | 59.10% | 1051 | 6884.1 | Pyro |
| 29 | chr9:5073770 | JAK2 | c.1849G>T | p.V617F | 41.30% | 512 | 1900.8 | Pyro |
| 30 | chr9:5073770 | JAK2 | c.1849G>T | p.V617F | 59.70% | 2823 | 13372.2 | Pyro |
|  | chr17:7578203 | TP53 | c.646G>A | p.V216M | 39.20% | 1734 | 5980.7 | Sanger |
| 31 | chr9:5073770 | JAK2 | c.1849G>T | p.V617F | 21.40% | 341 | 444.1 | Pyro |
|  | chr20:31024488 | ASXL1 | c.3973C>T | p.L1325F | 47.90% | 1213 | 5752.8 | n.d. |
| 32 | chr9:5073770 | JAK2 | c.1849G>T | p.V617F | 92.30% | 298 | 4074.8 | Pyro |
| 33 | chr9:5073770 | JAK2 | c.1849G>T | p.V617F | 41.10% | 1449 | 5353.5 | Pyro |
| 34 | chr9:5073770 | JAK2 | c.1849G>T | p.V617F | 62.40% | 749 | 5396.7 | n.d. |
|  | chr17:7578203 | TP53 | c.646G>A | p.V216M | 33.60% | 1382 | 3725 | Sanger |
| 35 | chr5:170837544 | NPM1 | c.860_863dupTCTG | p.W288C fs*12 | 43,7% | 3048 | 8111.2 | Sanger |
|  | chr1:115258744 | Nras | c.38G>A | p.G13D | 43,5% | 1077 | 4374 | Pyro |
|  | chr4:106190860 | TET2 | c.4140_4145delTGCCCA | p.A1381_H1382del | 89,6% | 27322 | 25117.9 | n.d. |
|  | chr2:25463242 | DNMT3a | c.2251T>G | p.F751V | 43,1% | 1357 | 5399.3 | n.d. |
|  | chr11:32413560 | WT1 | c.1390G>A | p.D464N | 39,8% | 1055 | 3697.8 | n.d. |
| 36 | chr9:5073770 | JAK2 | c.1849G>T | p.V617F | 62.10% | 175 | 1210.4 | Pyro |
|  | chr21:36206731 | RUNX1 | c.697_700delAACC | p.N233Lfs*50 | 50.00% | 836 | 2598 | n.d. |
| 37 | chr9:5073770 | JAK2 | c.1849G>T | p.V617F | 65.30% | 2381 | 15351.4 | Pyro |
|  | chr4:106158509 | TET2 | c.3410G>A | p.G1137D | 24.80% | 1986 | 3248.2 | n.d. |
|  | chr4:106190803 | TET2 | c.4081G>T | p.G1361C | 43.10% | 23513 | 8017.1 | n.d. |
|  | chr4:106197378 | TET2 | c.5711A>G | p.H1904R | 21.60% | 2886 | 2586.8 | n.d. |
|  | chr17:7577106 | TP53 | c.832C>A | p.P278T | 39.60% | 9784 | 7025.8 | Sanger |
| 38 | chr9:5073770 | JAK2 | c.1849G>T | p.V617F | 88.40% | 345 | 4384.8 | n.d. |
|  | chr2:25457243 | DNMT3a | c.2644C>T | p.R882C | 45.90% | 1330 | 5910.8 | Pyro |
| 39 | chr9:5073770 | JAK2 | c.1849G>T | p.V617F | 86.50% | 2647 | 24326.1 | Pyro |
|  | chr2:25457243 | DNMT3a | c.2644C>T | p.R882C | 43.70% | 4433 | 8227.9 | n.d. |
|  | chr20:31022643 | ASXL1 | c.2128G>T | p.G710* | 5.60% | 7213 | 141.7 | n.d. |
| 40 | chr9:5073770 | JAK2 | c.1849G>T | p.V617F | 61.40% | 2757 | 13979 | Pyro |
| 41 | 4:106,156,718 | TET2 | c.1620delT | p.L541* | 29.10% | 592 | 740.3 | n.d. |
