# Supplementary figures and images for: Comprehensive Molecular Profiling of Archival Bone Marrow Trephines Using a Commercially Available Leukemia Panel and Semiconductor-Based Targeted Resequencing
Source: PLoS One. 2015 Jul 29;10(7):e0133930. doi: 10.1371/journal.pone.0133930 (PMC4519100; doi:10.1371/journal.pone.0133930)

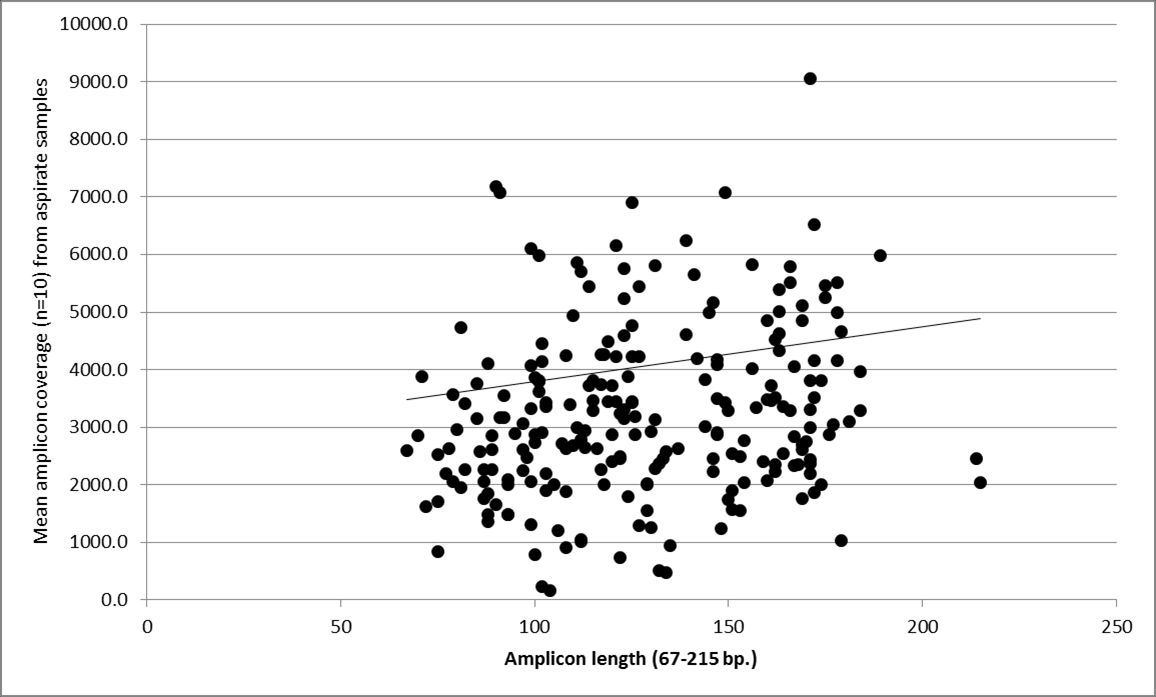

Supplement: S1 Fig — Eleven amplicons which obtained more than 10,000 reads are not shown. The very weak positive correlation is regarded by us as not real (Spearman r = 0.211, p = 0.0014; linear regression r2 = 0.0414). (TIF) [file pone.0133930.s005.tif]

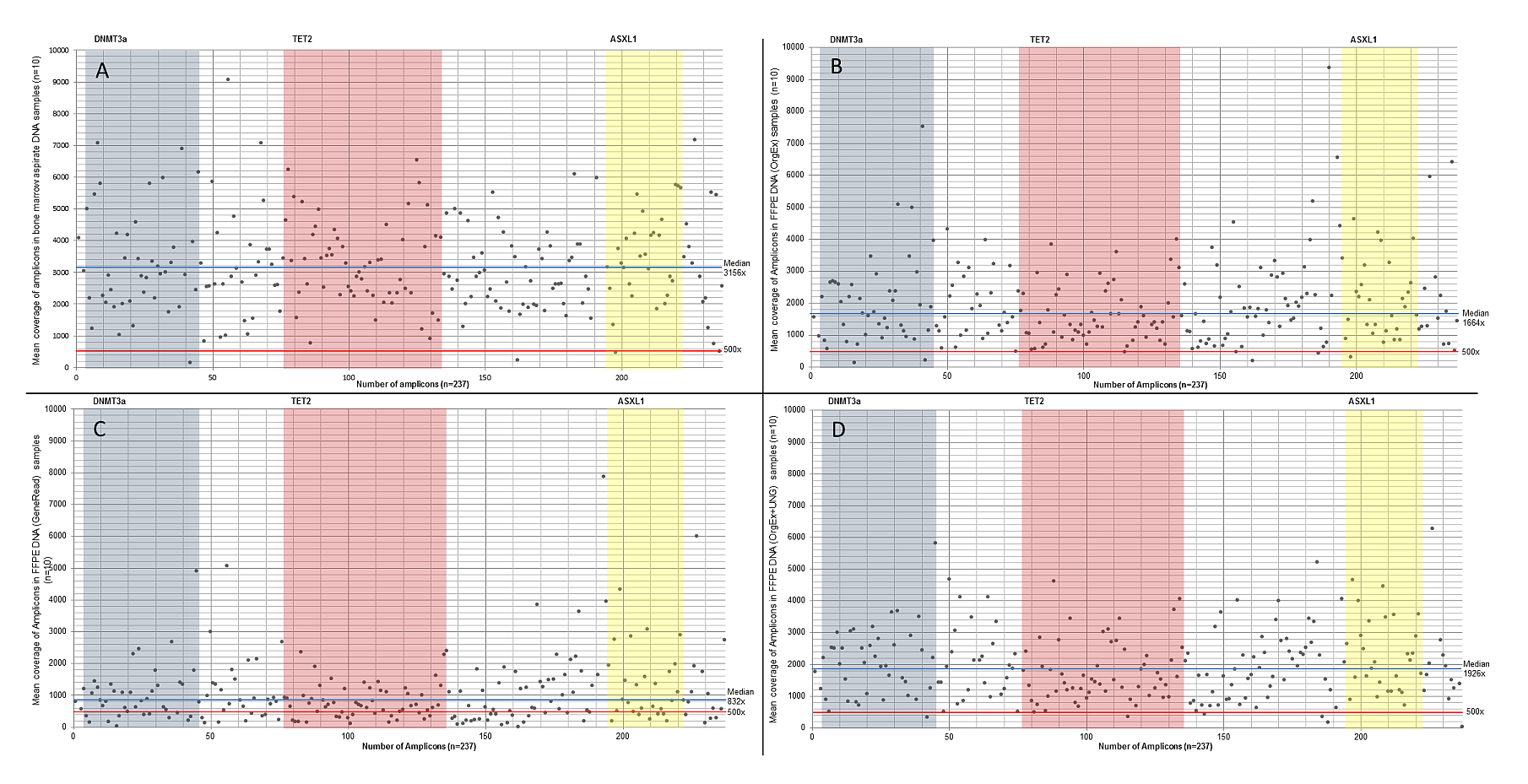

Supplement: S3 Fig — The uniformity of the mean sequencing coverage for all 237 amplicons of the AML Panel within the four sample sets (each n = 10) is displayed. (A) MAC of the aspirate DNA samples (11 amplicons with mean coverage >10.000x are not included: 1x NRAS, 2x DNMT3A, 1x GATA2, 2x TET2, 4x CEBPα, and 1x RUNX1). (B) MAC of the standard protocol FFPE DNA samples (7 amplicons with a mean coverage >10.000x are not included: 1x amplicon NRAS, 2x GATA2, 2x TET2, 1x CEBPα, and 1x RUNX1). (C) MAC of GeneRead Kit isolated FFPE DNA samples (12 amplicons with a mean coverage >10.000x are not included: 1x amplicon NRAS, 3x DNMT3A, 2x GATA2, 2x TET2, 2x CEBPα, and 2x RUNX1). (D) MAC of standard protocol + UNG pre-treatment FFPE DNA samples (16 with a mean coverage >10.000x are not included: 1x amplicon NRAS, 3x DNMT3A, 3x GATA2, 1x KIT, 2x TET2, 1x TP53, 2x CEBPα, and 3x RUNX1). All amplicons representing one of the “large” genes (i.e., DNMT3A, TET2, and ASXL1) are highlighted by a colored box (grey: DNMT3A, red: TET2, yellow: ASXL1). The median amplicon coverage over all amplicons is indicated by a horizontal blue line. The lower read depth threshold for reliable evaluation of 500 reads per amplicon is indicated by a red horizontal line. (TIF) [file pone.0133930.s007.tif]
